# Long-Term Symptoms of Mobile Phone Use on Mobile Phone Addiction and Depression Among Korean Adolescents

**DOI:** 10.3390/ijerph16193584

**Published:** 2019-09-25

**Authors:** So-Young Park, Sonam Yang, Chang-Sik Shin, Hyunseok Jang, So-Youn Park

**Affiliations:** 1 Ewha Institute for Age Integration Research, Ewha Womans University, 52, Ewhayeodae-gil, Seodaemun-gu, Seoul 03760, Korea; syp279@gmail.com; 2 Kyonggi University, San 94-6, Iui-dong, Yeongtong-gu, Suwon, Kyonggi-do 16227, Korea; snyang@kyonggi.ac.kr (S.Y.); hsjang@kyonggi.ac.kr (H.J.); 3 Daejeon University, 62, Daehak-ro, Dong-gu, Daejeon 34520, Korea; csshin@dju.kr

**Keywords:** mobile phone use, mobile phone addiction, depression, Korean adolescents

## Abstract

This study aimed to compare the mean scores of mobile phone use, mobile phone addiction, and depressive symptoms at three-time points among Korean adolescents according to gender and to examine the differences in the long-term relationships among the three abovementioned variables between Korean boys and girls in a four-year period. Data for 1794 adolescents (897 boys and 897 girls) were obtained from three waves of the second panel of the Korean Children and Youth Panel Survey. Multigroup structural equation modeling was used for data analyses. The study findings showed that at each of the three-time points, Korean girls tended to use their mobile phones more frequently and were at a higher risk of mobile phone addiction and depressive symptoms than Korean boys. Significant changes were observed in the longitudinal relationships among phone use, mobile phone addiction, and depressive symptoms in Korean adolescents across time periods, but no gender differences were found in the strengths of these relationships. These findings contribute to expanding the knowledge base of mobile phone addiction and depressive symptoms among Korean adolescents.

## 1. Introduction

Today’s adolescents are generally considered as the digital generation [1]. They have grown up using mobile phones, which have become an important part of their life and have reshaped their social life and behavior. Although mobile phones have many advantages such as the convenience of searching information, researchers have expressed concern regarding the potential negative effects of problematic mobile phone use, such as depression, anxiety, sleep disturbance, technostress, and poor academic performance [2,3]. In this regard, Korean adolescents have been found to be at considerable risk of mobile phone addiction. A recent national survey in South Korea in 2018 revealed that 29.3% of adolescents were dependent on mobile phones [4].

Various terms have been used to describe varying degrees of potential problems related to uncontrolled mobile phone use; the terms of problematic mobile phone use [5], mobile phone dependence [6], and mobile phone addiction [7] are used interchangeably. Although researchers have not reached a consensus regarding the definition of mobile phone addiction, potential indicators of mobile phone addiction include preoccupation with one’s mobile phone, conflicts with one’s family members resulting from the excessive use of the mobile phone, use of the mobile phone to handle changes in mood, and feeling of unease when mobile phone use is inhibited [7].

Researchers have identified gender differences in mobile phone use and mobile phone addiction [1,6,8]. Several studies have reported that female youth are likely to spend more time on their mobile phones than male youth [6,8], suggesting that females are potentially more vulnerable to mobile phone addiction than males [8]. In addition, gender differences have been found in the patterns of mobile phone usage. For example, female adolescents tend to use their mobile phones for text messaging, social media, for playing online games and for other forms of entertainment [6,8].

As regards the association between problematic mobile phone addiction and mental health issues, mobile phone addiction is consistently linked to depression [9]. Behavioral addiction such as internet addiction and mobile phone addiction during adolescence can be understood with a developmental psychopathology framework [10]. Adolescents go through the rapid development of emotional, social, and psychological changes, and, in particular, impulsive tendency is demonstrated during this time. Such impulsive behavior of the adolescent is intensified if the self-control is limited. This adolescent’s limited ability for self-control may be more vulnerable and susceptible to addictive behavior [11].

Most cross-sectional studies on mobile phone addiction have yielded inconclusive findings, but a few longitudinal studies have shown a bidirectional association between mobile phone addiction and depression [12,13]. For instance, Jun [12] investigated reciprocal effects between mobile phone addiction and depression using autoregressive cross-lagged modeling and determined the bidirectional associations between mobile phone addiction and depression over time. Specifically, significant reciprocal relationships were found over a period of three years, and both mobile phone addiction and depression became severe with time. On the other hand, Coyne Stockdale, and Summers [14] tested a bidirectional longitudinal model representing the relationship between depression and problematic mobile phone use and did not find any bidirectional relationship between them.

Considering these mixed findings on the directional relationships between mobile phone addiction and depression, we aimed to fill the gaps found in previous studies in a number of ways. In particular, the present study expanded on the earlier study by Jun [12], which showed the reciprocal association between mobile phone addiction and depression using the same Korean Children and Youth Panel Survey (KCYPS) data. First, this study included mobile phone use data, which were not included in Jun’s study [12], and examined the longitudinal associations among mobile phone use, mobile phone addiction, and depression. Several studies have found relationships between the frequency or duration of mobile phone use and mobile phone addiction [15,16], suggesting that an increase in mobile phone use causes mobile phone addiction. Second, the present study incorporated important factors related to family (i.e., parents’ education and income) and school (i.e., academic activity and relationships with peers), which were not controlled in Jun’s study [12]. Jun [12] mentioned that one of the limitations of his study was that it did not include relevant variables associated with mobile phone addiction and depressive symptoms. Third, considering that several studies found gender differences in various relationships, this study further investigated the role of gender in the longitudinal relationships among mobile phone use, mobile phone addiction, and depression in Korean youth.

Therefore, the purposes of this study were as follows: (1) to compare differences between Korean boys and girls regarding the mean scores of mobile phone use, mobile phone addiction, and depression across three time points, and (2) to investigate gender differences in the longitudinal relationships among mobile phone use, mobile phone addiction, and depression across different time periods.

## 2. Present Study

**Figure 1:**
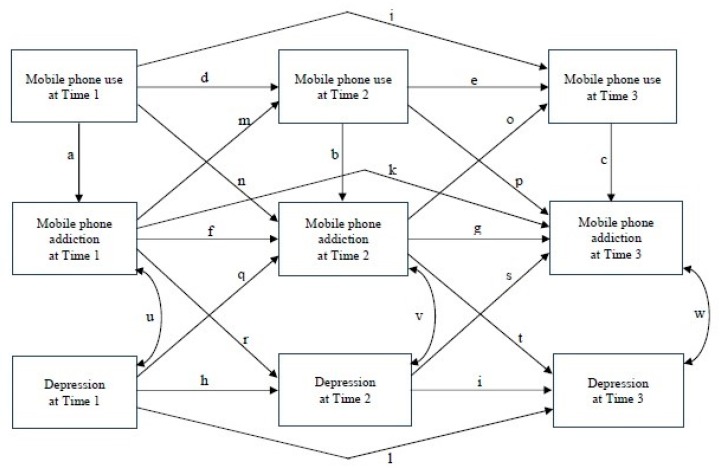
Structural model.

The structural model used in this study is displayed in Figure 1. The hypotheses are as follow:

**Hypothesis** **1** **(H1).** *The mean values of mobile phone use, mobile phone addiction, and depression for Korean girls will be higher than those for Korean boys in the second year of middle school (Time 1), first year of high school (Time 2), and third year of high school (Time 3).*

**Hypothesis** **2** **(H2).** *The longitudinal relationships among mobile phone use, mobile phone addiction, and depression will change from Time 1 to Time 3, and the strengths of these changes will differ between Korean boys and girls.*

H2a. There will be contemporaneous effects of mobile phone use on mobile phone addiction at each time point (Path a, Path b, and Path c).

H2b. There will be first-order autoregressive effects of mobile phone use (Paths d and e), mobile phone addiction (Path f and Path g), and depression (Path h and Path i) across time periods.

H2c. There will be second-order autoregressive effects of mobile phone use, mobile phone addiction, and depression (Path j, Path k, and Path l) from Time 1 to Time 3.

H2d. There will be cross-lagged effects between mobile phone use and mobile phone addiction (Path m, Path n, Path o, and Path p) and between mobile phone addiction and depression (Path q, Path r, Path s, and Path t) across time periods.

H2e. There will be bidirectional relationships between mobile phone addiction and depression at each time point (Path u, Path v, and Path w).

H2f. There will be gender differences in the hypotheses (H2a~H2e) stated previously.

## 3. Materials and Methods

### 3.1. Data Source and Sample

This study used the second panel of the KCYPS data collected by the National Youth Policy Institute (www.nypi.re.kr/archive) from 2010 to 2016. The KCYPS is a nationally representative longitudinal panel survey that repeatedly collects data of three cohorts of Korean children and adolescents. The sampling frame of the KCYPS was based on stratified multistage cluster sampling. Specific data collection procedures used in the KCYPS have been described in the literature [17].

The present study used the middle school student cohort data from the second wave (the year of 2011, 2nd grade in middle school, 14-year-old, Time 1), fourth wave (the year of 2013, 16-year-old, 1st grade in high school, Time 2), and sixth wave (the year of 2015, 3rd grade in high school, 18-year-old, Time 3) of the KCYPS. The observed sample at Time 1 consisted of a total of 2280 adolescents (1152 boys and 1128 girls). We included only those adolescents who had owned mobile phones in each time period when the data were collected. A final sample was 1794 adolescents (897 boys and 897 girls) [17].

This study was exempt from the Institutional Review Board (IRB) for the protection of human subjects (IRB No: 1040647-201810-HR-006).

### 3.2. Measures

Mobile Phone Use. The KCYPS assessed mobile phone use using a set of nine items [17]. Items assessed the frequency of mobile phone use by asking the respondents how often they used their mobile phones, such as ‘How often do you use your mobile phone to talk to your family?’ ‘How often do you use your mobile phone to text or message your friends?’ ‘How often do you use your phone to use game applications?’ Items were rated on a four-point Likert scale ranging from 1 (not at all) to 4 (very frequently). All the items required reverse coding, higher scores indicated higher levels of mobile phone use. The alpha coefficients for this measure in this study were 0.690 (Time 1), 0.703 (Time 2), and 0.706 (Time 3).

Mobile Phone Dependence. Mobile phone addiction was assessed according to a seven-item version of the Inventory for Mobile Phone Dependency (IMPD) [18]. It examines how much the respondents were addicted to their mobile phones, for instance: ‘I feel anxious without my mobile phone when I go out of the house,’ ‘I lose track of time when using my mobile phone,’ and ‘I cannot live without my mobile phone.’ The responses were rated on a four-point Likert scale ranging from 1 (never true) to 4 (always true), and all the items required reverse coding. Higher scores indicated greater levels of mobile phone dependency. Cronbach’s alpha coefficients in this study were 0.898 (Time 1), 0.884 (Time 2), and 0.867 (Time 3).

Depression. Depression was measured according to a shorter 10-item version of the Korean Manual of Symptom Checklist (KMSC) [19] such as ‘I have little energy,’ ‘I feel unhappy and depressed,’ ‘I have many worries,’ and ‘Everything is overwhelming for me.’ These items were rated on a four-point Likert scale ranging from 1 (never true) to 4 (always true), and all the items were reverse-scored. Higher scores indicated more severe depression. The alpha coefficients in this study were 0.901 (Time 1), 0.888 (Time 2), and 0.880 (Time 3).

Covariates. Covariates included factors related to family (i.e., two-income family, both parents living in the household, father’s education, and mother’s education, annual household income) and school (i.e., academic activity, compliance with school rules, and youth relationships with peers and teachers). Gender was used as a moderator.

The factor “two-income family” was dichotomized into two categories: both parents were working (1) and at least one parent was not working (0). The factor “both parents living in the household” was assessed by asking respondents whether or not they were living with both their parents, the responses were scored as either 1 (yes) or 0 (no). The annual household income was categorized into four categories as follow: 1 = less than $30,000, 2 = $30,000 to $40,000, 3 = $40,000 to $55,000, and 4 = greater than $55,000. The educational level of parents was scored as follows: 1 = less than high school, 2 = high school graduate, 3 = two-year college graduate, and 4 = four-year college graduate or above.

The School Adjustment Inventory was composed of 19 items with four subscales: academic activity, compliance with school rules, relationships with peers, and relationships with teachers [17]. The subscale “relationships with peers” consisted of four items, whereas the other three subscales included five items. The responses were rated on a four-point Likert scale ranging from 1 (never true) to 4 (always true). Higher scores indicated a greater level of school adjustment. Cronbach’s alpha coefficients for the four abovementioned subscales in this study were 0.696, 0.783, 0.677, and 0.835, respectively.

### 3.3. Strategy for Data Analyses

The family and school factors were descriptively examined for Korean boys and girls and compared using *t*-tests and chi-square tests. The *t*-tests were used to compute group mean comparisons of mobile phone use, mobile phone addiction, and depression in boys and girls at each time point.

A measurement invariance model was tested to ensure the metric equivalence of the three factors between boys and girls. Then, a multiple group structural equation modeling (MGSEM) approach was performed to compare gender group differences in unstandardized coefficients for the paths in Figure 1 [20]. First, an unconstrained model without equality constraints was estimated across the entire set of boys and girls. Second, a constrained model with equality constraints was separately estimated across groups. Then, a nested chi-square difference test was performed to compare the constrained and unconstrained models. Model fit was estimated using the following indices: the overall chi-square test (*p* > 0.05), the comparative fit index (CFI > 0.95), the root mean square error of approximation (RMSEA < 0.05), and the standardized root mean residual (SRMR ≤ 0.08) [21]. Missing data were dealt with using a full-information maximum likelihood approach [22]. Mplus 8.1 [23] and SPSS version 24.0 [24] were used for data analyses.

## 4. Results

### 4.1. Descriptive Analyses

Table 1 presents the group comparisons of family and school factors between Korean boys and girls in the second year of middle school (Time 1). Some significant differences between boys and girls were found for the school factor but not for the family factor. Among the school factors, girls reported higher levels of compliance with school rules (*t* = 2.94, *p* < 0.01) and relationships with peers (*t* = 3.80, *p* < 0.001).

### 4.2. Group Comparisons

Table 2 lists the group mean comparisons of mobile phone use, mobile phone addiction, and depression between boys and girls. Statistically significant group differences were found in the means of mobile phone use, mobile phone addiction, and depression at Times 1, 2, and 3. Mobile phone use for girls was higher than that for boys across time periods (Time 1: *t* = 7.25, *p* < 0.001, Time 2: *t* = 5.44, *p* < 0.001, Time 3: *t* = 4.94, *p* < 0.001). Similarly, girls showed higher mobile phone addiction than boys at each time point (Time 1: *t* = 8.20, *p* < 0.001, Time 2: *t* = 6.49, *p* < 0.001, Time 3: *t* = 5.80, *p* < 0.001). The means of the depression for girls were also higher than those for boys over time (Time 1: *t* = 6.12, *p* < 0.001, Time 2: *t* = 7.71, *p* < 0.001, Time 3: *t* = 7.50, *p* < 0.001). Therefore, hypothesis H1 was supported.

### 4.3. Structural Equation Model

Measurement invariance tests were performed for mobile phone use, mobile phone addiction, and depression among boys and girls using multiple-group confirmatory factor analyses. Among the 69 group comparisons, 15 paths linking from the unobserved variables to the observed indicators were statistically significant. These links represented 21.7% of all the group contrasts, which indicated that the path coefficients for the three factors largely measured the mobile phone use, mobile phone addiction, and depression in a similar manner between boys and girls. Thus, this study used latent composite variables to examine the structural model shown in Figure 1.

**Figure 2:**
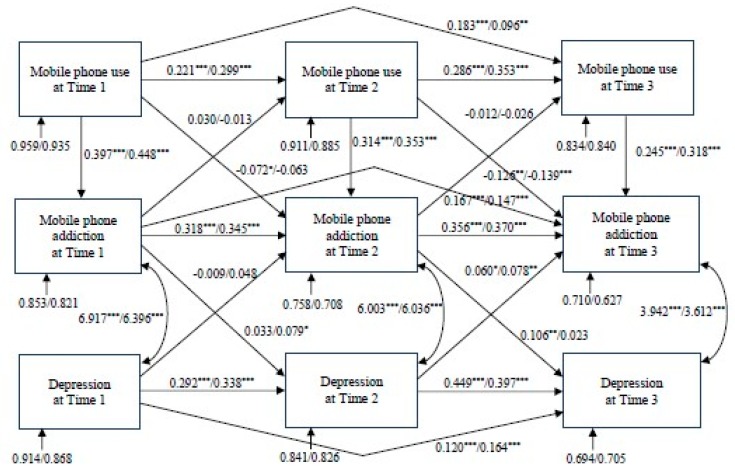
Autoregressive cross-lagged multiple-group structural equation model (Note: *** *p* < 0.001, ** *p* < 0.01, * *p* < 0.05. The first coefficient is the unstandardized path coefficient for boys, the second coefficient is the unstandardized path coefficient for girls. All coefficients are unstandardized path coefficients but disturbance values are standardized. Covariates include two-income family, both parents in the household, father’s education, mother’s education, annual household income, academic activity, compliance with school rules, and youth relationships with peers and teachers).

To test hypothesis 2, the structural model of mobile phone use, mobile phone addiction, and depression in boys and girls were tested using an MGSEM strategy with a Huber–White maximum likelihood estimator. The model fit was good (χ^2^(df = 26) = 33.826, *p* > 0.05, CFI = 0.998, RMSEA = 0.018, SRMR = 0.008, 90% C.I. = (0.000, 0.034)). Focused fit tests revealed no theoretically meaningful significant points of ill fit. Figure 2 shows the unstandardized path coefficient comparisons between boys and girls. For each of the two groups, statistically significant path coefficients were found for the contemporaneous effects of mobile phone use on mobile phone addiction at Times 1, 2, and 3 for boys (path coefficients = 0.397, 0.314, and 0.245, respectively) and girls (path coefficients = 0.448, 0.353, and 0.318, respectively). Therefore, hypothesis H2a was supported.

For the first-order autoregressive effects of mobile phone use, statistically significant effects were found for boys (Time 1 to Time 2: path coefficient = 0.221, Time 2 to Time 3: path coefficient = 0.286) and girls (Time 1 to Time 2: path coefficient = 0.299, Time 2 to Time 3: path coefficient = 0.353). Similarly, the first-order autoregressive effects of mobile phone addiction were observed for boys (Time 1 to Time 2: path coefficient = 0.318, Time 2 to Time 3: path coefficient = 0.356) and girls (Time 1 to Time 2: path coefficient = 0.345, Time 2 to Time 3: path coefficient = 0.370). For depression, the predicted first-order autoregressive effects from Time 1 to Time 2 and from Time 2 to Time 3 were observed for boys (path coefficients = 0.292 and 0.449) and Korean girls (path coefficients = 0.338 and 0.397). Additionally, the second-order autoregressive effects of mobile phone use, mobile phone addiction, and depression were found from Time 1 to Time 3 for boys (path coefficients = 0.183, 0.167, and 0.120) and girls (path coefficients = 0.096, 0.147, and 0.164). Therefore, hypotheses H2b and H2c were supported.

As for cross-lagged effects, statistically significant path coefficients were found between mobile phone use at Time 1 and mobile phone addiction at Time 2 for boys (path coefficient = 0.072) and between mobile phone use at Time 2 and mobile phone addiction at Time 3 for boys (path coefficient = −0.126) and girls (path coefficient = −0.139). However, no reciprocal causal relationships were found between mobile phone use and mobile phone addiction across time periods for both groups. Statistically significant path coefficients were found from mobile phone addiction at Time 1 to depression at Time 2 for girls (path coefficient = 0.079) and from mobile phone addiction at Time 2 to depression at Time 3 for boys (path coefficient = 0.106). Reciprocal causal relationships linking depression at Time 1 to mobile phone addiction at Time 2 were not observed, but reciprocal causal relationships linking depression at Time 2 to mobile phone addiction at Time 3 were found for boys (path coefficient = 0.060) and girls (path coefficient = 0.078). Therefore, hypothesis H2d was partially supported.

For bidirectional relationships, statistically significant path coefficients were found between mobile phone addiction and depression at each time point for boys (path coefficients = 6.917, 6.003, and 3.942) and girls (path coefficients = 6.396, 6.036, and 3.612). Therefore, hypothesis H2e was supported.

Finally, there were no gender differences for each path in the structural model. Thus, hypothesis H2f was not supported.

## 5. Discussion

This study aimed to investigate the gender differences in the mean scores of mobile phone use, mobile phone addiction, and depression among Korean youth and to compare differences in the longitudinal relationships of these three variables among boys and girls for a four-year period. The following four main points are discussed: (1) gender differences in the mean values of mobile phone use, mobile phone addiction, and depression, (2) longitudinal effects of mobile phone use, mobile phone addiction, and depression, (3) bidirectional relationships between mobile phone addiction and depression, and (4) gender differences in the structural model.

First, the mean values of mobile phone use, mobile phone addiction, and depression were differed by gender. Compared to boys, Korean girls were likely to use their mobile phones more and were at a higher risk of mobile phone addiction and depression during the second year of middle school (Time 1), the first year of high school (Time 2), and the third year of high school (Time3). These findings were consistent with those of a previous study by Sánchez-Martínez and Otero [6], which reported that female adolescents tended to use their mobile phones more often and were more likely to depend on mobile phone devices than their male counterparts. Similarly, girls were more likely to have depression than boys [25].

With regard to the autoregressive effects of mobile phone use, mobile phone addiction, and depression over time, the present study found that early mobile phone use, mobile phone addiction, and depression during middle school were associated with later mobile phone use, mobile phone addiction, and depression during high school. Frequent mobile phone users at middle school showed the tendency to use their mobile phones frequently even in high school. Similarly, Korean adolescents who showed higher levels of mobile phone addiction during the junior high school period were more likely to depend on their mobile phones during the high school period. This finding was consistent with previous studies [14,26], which indicated that mobile phone addiction tended to increase over time. As for depression, Korean adolescents with higher depression during the early adolescence period were at a higher risk of developing depression during the late adolescence period, which was consistent with the results of a previous study [25].

Regarding the cross-lagged effects of mobile phone use, mobile phone addiction, and depression, the present study found that the frequency of mobile phone use could not predict mobile phone addiction at a later time period. However, mobile phone addiction and depression concurrently predicted each other across time periods. First, frequent mobile phone users in middle school were less likely to addict to their mobile phones in high school. This finding was contrary to our hypothesis. The inconsistent finding might be attributable to the use of different measurement tools. Haug et al. [27] found that smartphone addiction was more strongly associated with the total time spent per day using smartphones than the frequency of smartphone use. In contrast, Lin et al. [28] reported that the frequency of mobile phone use was an indicator of smartphone addiction rather than the total time spent using mobile phones. These two studies [27,28] used both the frequency of mobile phone use and the total time spent using mobile phones and mainly carried out cross-sectional analysis. The present study did not include the variable of the total time spent using mobile phones but longitudinally analyzed the relationship between the frequency of mobile phone use and mobile phone addiction. Therefore, further longitudinal study is warranted to examine the relationships among the total time spent using mobile phones, the frequency of mobile phone use, and mobile phone addiction. Second, earlier depression in the first year of high school (Time 2) was associated with later mobile phone addiction in the third year of high school (Time 3). This finding supports that of a previous study, which indicated that depression was a predictor of mobile phone addiction [29]. Third, this present study found that earlier mobile phone addiction was associated with later depression. This finding, that supported the study by Coyne et al. [14], indicated that early problematic mobile phone use was a significant predictor of depression.

This study confirmed that the bidirectional relationship between mobile phone addiction and depression persisted even after the other variables were controlled. This finding is consistent with Jun’s study [12]. There are some possible explanations for this causal pathway. Adolescents with depression are more likely to be addicted to their mobile phones because mobile phones may provide an environment where such adolescents can relate to others in a safer and less demanding environment [6]. Inversely, a possible explanation of why mobile phone addiction may lead to depression is that adolescents with higher levels of mobile phone addiction might be at an increased risk of interpersonal problems, resulting in depression later [30]. This finding highlights the importance of providing simultaneous intervention for reducing both depression and mobile phone addiction.

Finally, contrary to our expectation, the present study found no gender differences for each path in the structural model. A plausible explanation for this finding is that the sample considered in this study consisted of middle and high school Korean students. In particular, high school students have less time to use their mobile phones than young adults because high school students need to prepare for university entrance exams. This commonality of adolescent life in South Korea may result in there being no gender differences. Although the present study analyzed data longitudinally, four years of research might not be sufficient to investigate gender differences in the relationships among mobile phone use, mobile phone addiction, and depression. Prior literature reported that gender was an important predictor in the relationship between the internet or mobile phone use and mental health outcomes but these studies were cross-sectional [1,31]. Few studies have explored the causal effects of gender on this issue, thus, more studies are needed to conduct with longitudinal data.

This study has some limitations. First, considering the nature of the secondary analysis, the present study was limited by the measurement of mobile phone use. The KCYPS did not measure the actual time and frequency of mobile phone use, which may yield different results regarding the relationship between mobile phone use and mobile phone addiction. A further longitudinal study is warranted to examine the relationships among the total time spent using mobile phones, the frequency of mobile phone use, and mobile phone addiction. Second, our study did not distinguish between different types of mobile phone devices but treated them (from the most advanced smartphones to standard cell phones) as the same. Thus, the findings of this study should be interpreted with caution. Future research is warranted to investigate whether the types of mobile phone devices are related to adolescents’ patterns of mobile phone use. Third, although we found significant associations among mobile phone use, mobile phone addiction, and depression over time in this study, causal inferences among these variables are limited due to the inconsistent results on this topic. Lastly, although this study included parents’ socio-economic variables such as income and education, it did not include parents’ psychological status such as mobile phone use of parents, which may affect their children’s mobile phone use. Future research is needed to examine the effect of parents’ psychological status on their children’s mobile phone use and mobile phone addiction.

## 6. Conclusions

In summary, excessive mobile phone use and mobile phone addiction have been increasing concerns among Korean adolescents. Findings of this study revealed that Korean girls were more exposed to mobile phone use and they were at higher risk of mobile phone addiction and depressive symptoms. In addition, the longitudinal relationships among mobile phone use, mobile phone addiction, and depressive symptoms were observed in Korean adolescents. This study implies that it is necessary to acknowledge the negative effects of mobile phone use and to design adequate intervention strategies to prevent mobile phone addiction and depressive symptoms among adolescents.

## Figures and Tables

**Table 1:** Group comparisons of family and school factors between boys and girls at Time 1 (*N* = 1794).

Variable	Boys (n = 897)		Girls (n = 897)		*t*/χ^2^
	Frequency (%)	*M* ± *SD*	Frequency (%)	*M* ± *SD*	
**Family factor**					
Two-income Family					
Yes	492 (54.8)		509 (56.7)		0.65
No	405 (45.2)		388 (43.3)		
Both parents in household					
Yes	766 (87.7)		772 (89.9)		1.97
No	107 (12.3)		87 (10.1)		
Annual household income ($)					
0–30,000	283 (33.4)		258 (30.7)		5.31
30,000–40,000	193 (22.8)		177 (21.1)		
40,000–55,000	168 (19.8)		204 (24.3)		
≥55,000	203 (24.0)		201 (23.9)		
Father’s education					
Less than high school	28 (3.5)		29 (3.6)		1.06
High school graduate	356 (44.2)		338 (42.1)		
Two-year college graduate	76 (9.5)		85 (10.6)		
Four-year college graduate or Above	344 (42.8)		351 (43.7)		
Mother’s education					
Less than high School	22 (2.7)		29 (3.5)		2.01
High school graduate	463 (57.7)		455 (55.3)		
Two-year college graduate	83 (10.3)		80 (9.7)		
Four-year college graduate or Above	235 (29.3)		259 (31.5)		
**School factor**					
Learning activity		13.64 ± 2.64		13.72 ± 2.46	0.68
Compliance with school rules		13.81 ± 2.87		14.18 ± 2.54	2.94 **
Youth relationships with peers		12.11 ± 2.04		12.45 ± 1.73	3.80 ***
Youth relationships with teachers		14.12 ± 3.34		13.95 ± 3.24	1.06

Note: *** *p* < 0.001, ** *p* < 0.01. *M* = mean, *SD* = standard deviation.

**Table 2:** Group mean comparisons of major variables between boys and girls (*N* = 1794).

Variable	Boys (n = 897)	Girls (n = 897)	*t*
	*M* ± *SD*	*M* ± *SD*	
2nd Grade of middle school at Time 1 (14-year-old)			
Mobile phone use	27.68 ± 4.27	29.04 ± 3.63	7.25 ***
Mobile phone addiction	15.07 ± 4.97	17.06 ± 5.32	8.20 ***
Depression	18.36 ± 5.94	20.09 ± 6.02	6.12 ***
1st Grade of high school at Time 2 (16-year-old)			
Mobile phone use	29.43 ± 4.08	30.40 ± 3.50	5.44 ***
Mobile phone addiction	15.98 ± 4.53	17.42 ± 4.89	6.49 ***
Depression	17.79 ± 5.34	19.78 ± 5.60	7.71 ***
3rd Grade of high school at Time 3 (18-year-old)			
Mobile phone use	29.57 ± 4.01	30.47 ± 3.70	4.94 ***
Mobile phone addiction	15.68 ± 4.51	16.91 ± 4.46	5.80 ***
Depression	17.75 ± 5.41	19.65 ± 5.34	7.50 ***

Note: *** *p* < 0.001. *M* = mean, *SD* = standard deviation.
